# Cutting Edge Research for Exploration of Biomolecules for Gemcitabine-Based Chemo-Resistant Advanced Bile Duct Cancer: From Basic Study to Clinical Trial

**DOI:** 10.3390/biom11111626

**Published:** 2021-11-03

**Authors:** Chiao-En Wu, Chun-Nan Yeh

**Affiliations:** 1 Division of Hematology-Oncology, Department of Internal Medicine, Chang Gung Memorial Hospital, Linkou Branch, Chang Gung University College of Medicine, Taoyuan 333, Taiwan; jiaoen@gmail.com; 2 Department of General Surgery and Liver Research Center, Chang Gung Memorial Hospital, Linkou Branch, Chang Gung University, Taoyuan 333, Taiwan

Bile duct cancer (BDC) has been identified as a highly aggressive cancer arising from epithelial cells of the bile duct, including intrahepatic, perihilar, and extrahepatic [1]. Together with gallbladder and periampullary cancer, these are collectively grouped into BDC. As patients with early BDC are often asymptomatic, BDCs are usually diagnosed at an advanced stage with poor prognosis or high recurrence rate after primary operation. Recently, BDC has become the focus of increasing concern to seek greater mechanistic insights and more effective biomarker-driven targeted approaches for managing and/or preventing this challenging cancer, largely due to its rising incidence and high mortality rates worldwide, particularly in Asian countries [1].

Palliative chemotherapy with cisplatin and gemcitabine (GEM) has been the standard of care for patients with unresectable cancers since 2010 and no breakthrough progress in this challenging disease in the era of targeted therapy and immunotherapy [2]. In order to serve as a reference for further research to overcome GEM-resistance and provide novel treatment strategies, this Special Issue entitled “Cutting-Edge Research for Exploration of Biomolecules for Gemcitabine-Based Chemoresistant Advanced Bile Duct Cancers: From Basic Study to Clinical Trial” aimed to integrate the cutting-edge research and expand knowledge on a wide range of topics, including bioinformatics [3], clinical trials [4], p. 53 [5], micro-RNA [6], and other associated studies [7].

In recent years, scientists have established many omics profiles to reveal underlying mechanisms and networks in cancer. This omics-based approaches can be used and integrated for further basic and clinical research. Chang et al. reviewed the integration of genomics, epigenomics, transcriptomics, proteomics and metabolomics in BDC and provided the views of the application of omics in tumorigenesis, identification of prognostic factors, and investigation of novel targets for current and future drug development [3].

One famous tumor suppression gene, *TP53* encoding p53 protein, the guardian of the genome [8], has been investigated for more than 40 years and plays crucial roles in cancers. Unfortunately, no treatment targeting p53 pathway has be approved nowadays but lots of compounds have been studied in preclinical research and clinical trials. Wu et al. reviewed the roles of p53 in GEM resistance and envisioned the future therapeutic strategies by targeting p53 in BDCs [5]. Other than p53, Chiang et al. reviewed the potential targeted therapy based on tumor genomic profiling such as FGFR2 fusion, IDH1 mutation, NTRK fusion, BRAF mutation etc. and provided therapeutic options after intolerance or failure to GEM-based chemotherapy [4].

Emerging evidence demonstrated that miRNAs regulate tumor responses to chemotherapy and targeted therapy. Huang et al. provided an overview of the current knowledge about the miRNA-mediated regulatory mechanisms underlying drug resistance among BDC, discussed the application of miRNA-based therapeutics to BDC, and provided the basis for innovative treatment approaches [6]. Pan et al. elucidated ATM inhibitors in GEM-resistant BDC cell lines through polymerase θ deficiency and provided ATM acting as a potential target [7]. Based on the defect on DNA repair pathway, synthetic lethality occurred while ATM was suppressed in GEM-resistant BDC cell lines.

Based on the comprehensive reviews and studies of various aspects in BDC from this special issue, to elucidate the successful strategy to overcome GEM resistance and innovate future novel treatment could be envisioned. All the efforts on BDC will help clinicians and researchers to beat such tough disease in the future.

## Data Availability

Not applicable.

## References

[1] Banales J.M., Marin J.J.G., Lamarca A., Rodrigues P.M., Khan S.A., Roberts L.R., Cardinale V., Carpino G., Andersen J.B., Braconi C. (2020). Cholangiocarcinoma 2020: The next horizon in mechanisms and management. Nat. Rev. Gastroenterol. Hepatol..

[2] Valle J., Wasan H., Palmer D.H., Cunningham D., Anthoney A., Maraveyas A., Madhusudan S., Iveson T., Hughes S., Pereira S.P. (2010). Cisplatin plus gemcitabine versus gemcitabine for biliary tract cancer. N. Engl. J. Med..

[3] Chang Y.C., Chen M.H., Yeh C.N., Hsiao M. (2020). Omics-Based Platforms: Current Status and Potential Use for Cholangiocarcinoma. Biomolecules.

[4] Chiang N.J., Chen L.T., Shan Y.S., Yeh C.N., Chen M.H. (2021). Development of Possible Next Line of Systemic Therapies for Gemcitabine-Resistant Biliary Tract Cancers: A Perspective from Clinical Trials. Biomolecules.

[5] Wu C.E., Pan Y.R., Yeh C.N., Lunec J. (2020). Targeting P53 as a Future Strategy to Overcome Gemcitabine Resistance in Biliary Tract Cancers. Biomolecules.

[6] Huang W.K., Yeh C.N. (2020). The Emerging Role of MicroRNAs in Regulating the Drug Response of Cholangiocarcinoma. Biomolecules.

[7] Pan Y.R., Wu C.E., Yeh C.N. (2020). ATM Inhibitor Suppresses Gemcitabine-Resistant BTC Growth in a Polymerase theta Deficiency-Dependent Manner. Biomolecules.

[8] Lane D.P. (1992). Cancer. p53, guardian of the genome. Nature.

